# Equine Sarcocystosis in the Northern Region of the Republic of Kazakhstan

**DOI:** 10.3390/ani14162299

**Published:** 2024-08-07

**Authors:** Zhanaidar Bermukhametov, Kulyay Suleimanova, Oksana Tomaruk, Bakhit Baimenov, Pavel Shevchenko, Assylbek Batyrbekov, Zoja Mikniene, Ahmet Onur Girişgin, Raushan Rychshanova

**Affiliations:** 1 Research and Innovation Center, Research Institute of Applied Biotechnology, NLC «Akhmet Baitursynuly Kostanay Regional University», Kostanay 110000, Kazakhstan; zhanaidar007@gmail.com (Z.B.); lebleucura@gmail.com (O.T.); bahytbajmenov@gmail.com (B.B.); pavel87011339688@gmail.com (P.S.); asylbek55555@gmail.com (A.B.); 2 Department of Natural Sciences, Kostanay Social-Technical University named after Academician Zulharnai Aldamjar, Kostanay 110000, Kazakhstan; sulejmanovakulaj@gmail.com; 3 Large Animal Clinic, Lithuanian University of Health Science, Veterinary Academy, LT-44307 Kaunas, Lithuania; zoja.mikniene@lsmuni.lt; 4 Department of Parasitology, Veterinary Faculty, Bursa Uludag University, 16059 Nilüfer-Bursa, Turkey; onurgirisgin@gmail.com

**Keywords:** sarcocystosis, horses, muscle, histological studies, molecular genetic analysis

## Abstract

**Simple Summary:**

Horse meat consumption in Kazakhstan is a long-standing tradition, dating back to the steppe nomads’ era. It has been a staple in the country’s culinary heritage for centuries. The issue of the safety and nutritional value of horse meat remains a significant concern. Our research has revealed the presence of sarcocystosis in horse meat in northern Kazakhstan. Sarcocystosis affects the muscle tissue, thereby reducing the meat nutritional value. The conducted analyses results demonstrated the consequences that parasites have on the muscles of affected animals, which inevitably affect the meat quality. Molecular genetic analyses have identified two species of the aforementioned parasite. It is important to note that sarcocystosis is a dangerous disease for humans. In immunodeficient individuals, it can lead to the development of various pathological processes and, in some cases, to a lethal outcome. The findings of our investigation elucidated the prevalence of sarcocystosis in horses. The data obtained will facilitate the formulation of a plan for the implementation of preventive measures to combat sarcocystosis and the dissemination of information among veterinary professionals employed on farms. This will undoubtedly contribute to the enhancement of safety and quality control measures for meat derived from domestic animals, while simultaneously preventing human infection.

**Abstract:**

Background: A total of 396 samples were taken from the hearts, oesophagi, and diaphragms of 132 horses slaughtered at slaughterhouses in 2023 for subsequent examination. Methods: The histological method revealed pathomorphological changes in the muscle tissue. The molecular method identified the pathogen species. Results: Histological examination revealed thick-walled cysts with internal septa and numerous bradyzoites, and mononuclear inflammatory cells with pericyst infiltrates. Microcyst samples were amplified by polymerase chain reaction. Molecular genetic analysis allowed for the identification of 18 sarcocysts. Phylogenetic analysis of *Sarcocystis* isolates revealed three separate clades of *Sarcocystis bertrami* and two separate clades of *Sarcocystis fayeri*. Comparison and phylogenetic analysis revealed a very close relationship between the identified *Sarcocystis* species and other equine *Sarcocystis* DNA sequences from China and Japan. Based on the results obtained, the epizootic situation and the parasitic level of sarcocystosis contamination of horses in the northern Kazakhstan were determined. Conclusion: This is the first histological and molecular study to identify *Sarcocystis* spp. isolated from microscopic forms of equine sarcocysts in the northern Kazakhstan. This research will contribute to the fight against the spread of sarcocystosis in the Republic of Kazakhstan and will allow us to develop proposals for improving the mechanisms of sarcocystosis control.

## 1. Introduction

The problem of food safety is the most important state and social priority, aimed at preserving and improving public health and the production of high-quality and safe products. Food products containing pathogenic bacteria, viruses, and parasites are the cause of many diseases [1,2,3]. Nowadays, the issue of obtaining quality meat, including horse meat, is of great importance [4,5]. One of the most dangerous diseases is sarcocystosis caused by protozoa of the genus *Sarcocystis*, which also affects horses [5,6].

Sarcocystosis is a poorly studied chronic zooanthroponotic invasive disease caused by protozoa of the genus *Sarcocystis*. It is characterised by the formation of cysts in transverse striated muscles and other tissues in a wide range of species, including mammals, birds and reptiles. The disease causes significant economic damage, including the delayed growth and development of young animals, reduced meat quality and productivity, and the death of sick animals. According to researchers from various countries worldwide, sarcocystosis is a prevalent and widespread disease, with a high prevalence of sarcocysts in adult animals [5,6,7,8,9,10,11].

Kazakhstan is the ninth largest country in the world, with more than 180 million hectares of pasture land, of which only 45% is used for its intended purpose. Due to the rearing of animals on permanently restricted contaminated pasture areas, infestation of animals with invasive elements occurs. Sarcocystosis is of epidemiological importance as it is dangerous for humans. The consumption of horse meat in food is part of the national character; the local people consume a lot of horse meat [11,12]. A person consuming insufficiently cooked meat becomes infected with the cysts of the parasite, resulting in intestinal sarcocystosis [13,14,15,16,17]. The pathogen, by invading and parasitising the tissues of vital organs, releases sarcotoxin and poses a threat to human and animal health [17]. To date, it is not known how many species of *Sarcocystis* can infect the horse. The horse is an intermediate host of the highly pathogenic species S.neurona, which causes central nervous system disease and specifically causes the protozoal disease equine myeloencephalitis [18,19].

A prerequisite for this research was the absence of contemporary studies concerning the dissemination of equine sarcocystosis in the northern region of the Republic of Kazakhstan. 

Due to insufficient study of the problem, the gaps and inconsistencies in the current legislation of the country on the prevention and control of this pathology, and the wide spread, high social significance of sarcocystosis, the aim of our work was to study the epizootic situation in the northern region of the Republic of Kazakhstan.

## 2. Materials and Methods

This study was approved by the Local Ethical Committee on Animal Use of Akhmet Baitursynuly Kostanay Regional University under the case number IBR00014274 (protocol number 6 approved 1 November 2022).

### 2.1. Muscle Tissue Samples

The material for research comprised samples of muscle tissue from horses aged between two and ten years old, belonging to the local steppe breed. The samples were selected at slaughterhouses in the Kostanay region during 2023. A mixed system of keeping is applied depending on the natural–climatic features of the region. Accordingly, during the warmer months, the horses are permitted to graze naturally; whereas during the colder months, they are kept in stables, where their diet consists of hay, oats, and water. A total of 132 horse carcasses were subjected to a pre-slaughter examination, comprising 80 stallions and 52 mares. From the carcasses, 396 samples of muscle tissue were obtained from the heart, oesophagus, and legs of the diaphragm, with a maximum weight of 50 g. 

### 2.2. Histological Studies

In order to perform histological studies, a sample of 15 muscle tissue samples with a high degree of invasion was obtained. Muscle tissue samples were examined for the presence of *Sarcocystis* spp. using a Leica LM 4000 light microscope. This involved viewing paraffin sections (5 µm) after fixation in 4% neutral formaldehyde solution. The material was then sealed by pouring it into paraffin using a MC-2 luge microtome. The thickness of the slices was 5–7 µm. The structure of muscle tissue was studied using haematoxylin and eosin, polychrome, and Van Gizon staining [20,21,22].

### 2.3. Molecular Analyses

The identification of species was conducted via polymerase chain reaction (PCR), targeting the mitochondrial cytochrome c oxidase gene (cox1). The DNA extraction of microbiologically identified sarcocysts was conducted using PureLink™ Genomic DNA Kits (Thermo Fisher Scientific, Carlsbad, CA, USA) in accordance with the manufacturer’s instructions.

To obtain further molecular identification, native, muscle-fibre-free *Sarcocystis* isolates were isolated from muscle fibres using prepore needles under a microscope. 

Single sarcocyst DNA was extracted using PureLink™ Genomic DNA Kits from Thermo Fisher Scientific. The mitochondrial cytochrome c-oxidase subunit 1 gene (cox1) was amplified using a set of primers (SF1-SR9, SF1-SR4, SF1-SR5, SF1-COIRm, SF1-CoxS1R) designed to target this specific region (Table 1) [23,24].

A polymerase chain reaction (PCR) was conducted in a 30 µL reaction mixture containing 15 µL of PCR Master Mix (2X), 3 µL (10 pM) of forward and reverse primers, 5 µL of water, and 7 µL of DNA matrix.

The following cycling conditions were employed: an initial hot start at 95 °C for 15 min, followed by 45 cycles of denaturation at 94 °C for 30 s, annealing at 52 °C for 30 s, and extension at 72 °C for 1 min and 30 s, with a final extension at 72 °C for 10 min. All polymerase chain reaction (PCR) reactions were conducted on an Applied Biosystems SimpliAmp instrument. The amplification results were then analysed via visual inspection on a 1.5% agarose gel.

PCR purification of amplification products was conducted using ExoSAP-IT™ reagent (Thermo Fisher Scientific, USA). Sequencing was conducted using the BigDye Terminator v3.1 Kit (Thermo Fisher Scientific, USA), following the manufacturer’s instructions. The purification of the sequencing reaction products was conducted using BigDye XTerminator (Thermo Fisher Scientific, USA). The samples were subjected to separation on an Applied Biosystems 3500 Genetic Analyzer (Thermo Fisher Scientific, USA). The resulting gene sequences were analysed using the Unipro UGENE tool and compared with sequences of closely related organisms deposited in GenBank™ using the BLAST tool.

Phylogenetic analysis was performed using the maximum cumulative likelihood model [25]. Evolutionary analysis was performed in MEGA11 [26,27].

## 3. Results

### 3.1. Histological Studies

All horses entered for slaughter were clinically healthy. The muscles of the heart, oesophagus and diaphragm were examined macroscopically, and no cysts were observed. Sarcocysts were identified in the muscles of horses examined via microscopy, with a prevalence particularly evident in the muscles of the heart, oesophagus, and diaphragm.

**Figure 1:**
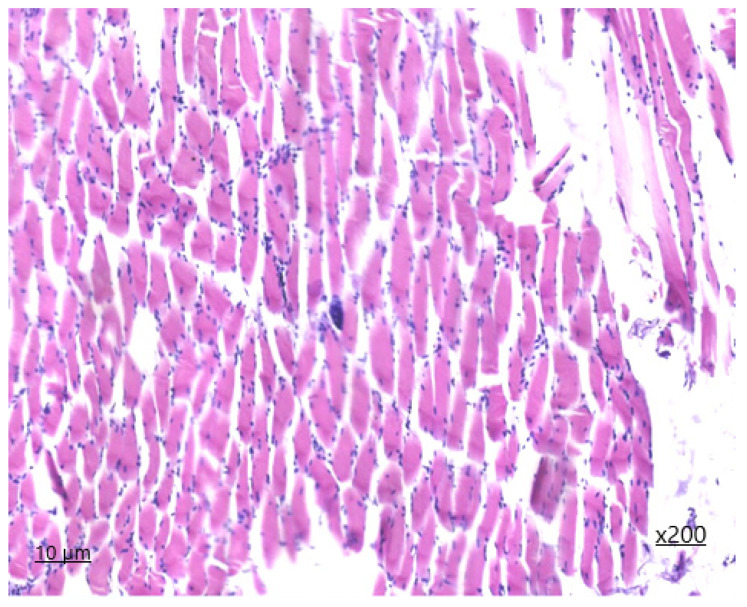
*Sarcocystis* in the cardiac tissue, first-generation staining with haematoxylin and eosin ×200.

**Figure 2:**
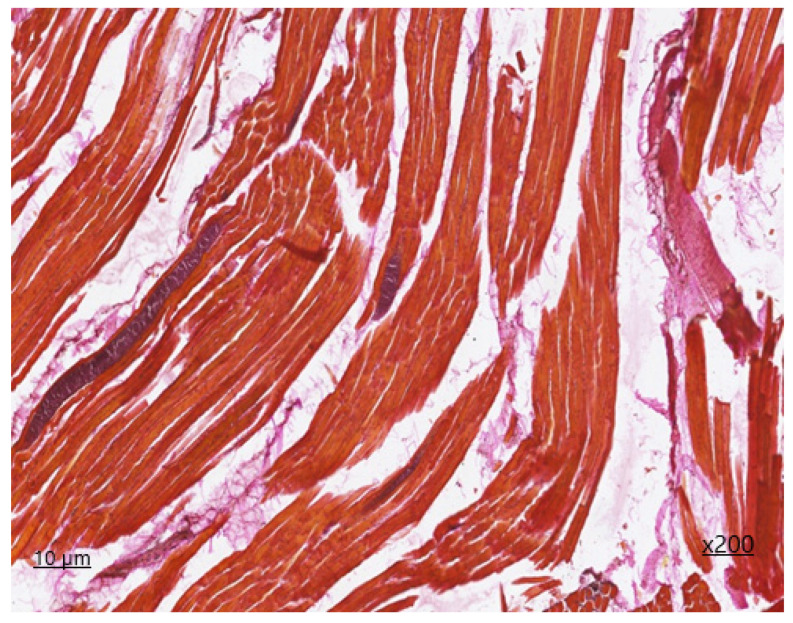
*Sarcocystis* in the cardiac tissue, second-generation Van Gieson staining ×200.

Upon histological examination of muscle sections from the heart (Figure 1 and Figure 2), it is evident that both longitudinal and partially transverse sections of muscle fibres are visible within the field of view. The ratio of muscle to adipose tissue is 90/1. Epimysium is not defined. The perimysium is of normal thickness and structure. The density of vessels is increased due to the presence of areas of neoangiogenesis and single nerve trunks. The density of endomysial slits is increased, containing a large number of capillary lumina and fibroblast nuclei, with areas of sprouting fibrous fibres. The sarcoplasm is slightly thickened, with muscle fibre nuclei located parallel to the periphery of the longitudinal line of the muscle fibre. The transverse striation in the unaffected muscle fibres is preserved, and the myoglobin saturation is sufficient. The diameter of the muscle fibre is 45 μm. On histological examination, the area of the lesion containing sarcocysts, predominantly of the second generation, is identified to be filled with endozoites, measuring 62 × 34 microns in size.

The affected muscle fibers display a lack of transverse striation, and the sarcoplasm forms an oval-shaped connective tissue capsule. On the periphery of the cyst capsule, non-specific inflammation is observed in the form of granulocytic infiltration with the presence of eosinophils. The degree of inflammation of the muscle tissue in the surrounding area is visualised in the form of serous reactive myositis of focal character. 

Upon additional histochemical staining by Van Gizon, the fibrous fibers exhibited a bright crimson colouration, with an increased density due to concentric structures on the periphery of neurovascular bundles and due to thickening of perimysium fibers deeply embedded in endomysial spaces. The reaction of muscle fibers is greenish-brown, indicating the absence of fibrous tissue within the muscle fiber. 

**Figure 3:**
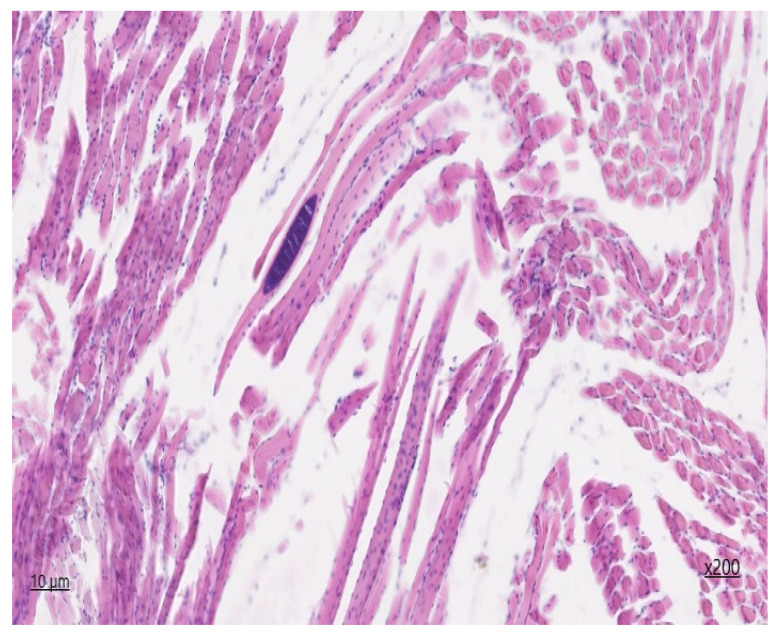
*Sarcocystis* in the oesophagus, second-generation meronth staining with haematoxylin and eosin ×200.

**Figure 4:**
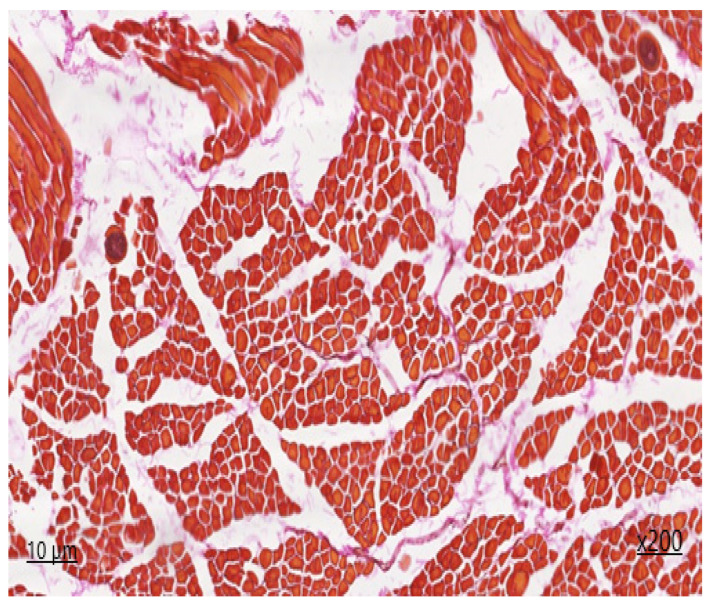
*Sarcocystis* in the oesophagus, second-generation merontium Van-Gizon staining ×200.

In the preparation of the oesophagus stained with hematoxylin and eosin (Figure 3) and Van-Gizon (Figure 4), a slice of transverse striated skeletal muscle tissue is identified. In the majority of cases, longitudinal and partially transverse sections of muscle fibers are identified. 

The perimysium is thickened as a result of the proliferation of connective tissue components, which is indicative of fibrosclerosis. Single nerve trunks are identified, and the density of vessels is augmented in areas of neoangiogenesis. The density of endomysial gaps is increased, as is the number of fibroblast nuclei. Additionally, multiple areas of fibrous fibre sprouting are detected. The sarcoplasm is slightly thickened, with muscle fibre nuclei located parallel to the periphery of the longitudinal line of the muscle fibre. In unaffected muscle fibres, transverse striation is preserved, and myoglobin saturation is sufficient. The diameter of the muscle fibres reaches up to 45 μm. In the histological section, areas of lesions with sarcocysts are observed, with an average of four cysts present. The affected muscle fibres display a lack of transverse striation, and the sarcoplasm forms a connective tissue capsule of varying shapes, including lemon-shaped, oval, and rounded. Second-generation meronts, which are filled with endozoites measuring 103 × 52 µm in size, are also present. On the periphery of the cyst capsule, a non-specific inflammatory response is observed, characterised by the presence of a histiocytic component with an admixture of lymphocytes. The degree of inflammation of the muscle tissue at a distance from the lesion area is visualised in the form of chronic myositis of focal character. In histochemical Van-Gizon staining, fibrous fibres exhibit a bright crimson hue, with increased density and occurrence due to concentric structures on the periphery of neurovascular bundles and due to the thickening of perimysium fibres deeply embedded in endomysial spaces. 

**Figure 5:**
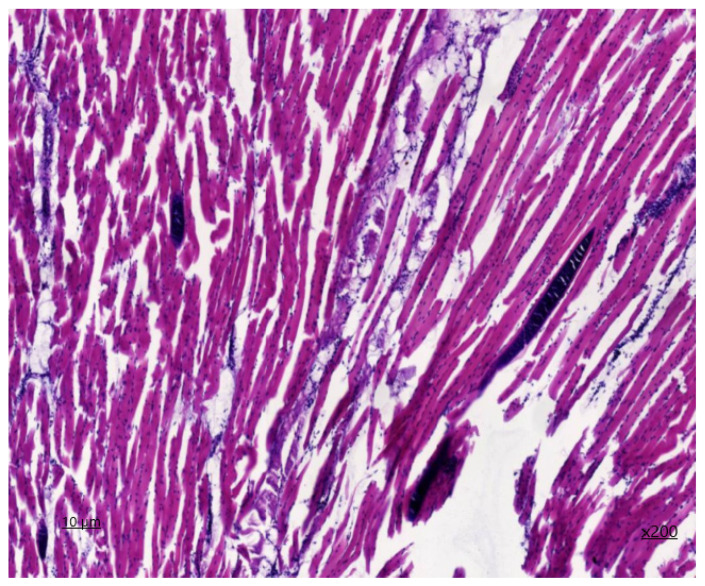
Sarcocysts in the diaphragm, haematoxylin and eosin staining ×200.

**Figure 6:**
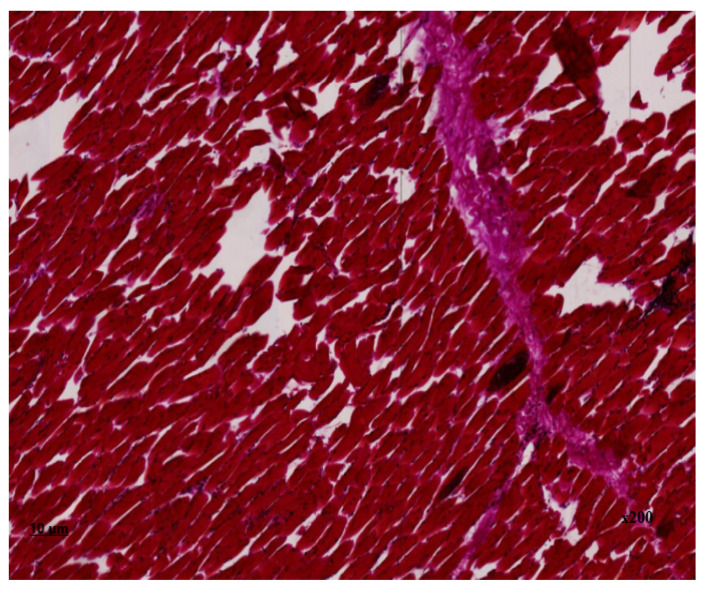
Sarcocysts in the diaphragm Van Gieson staining ×200.

In the preparation of the legs of the diaphragm (Figure 5 and Figure 6), longitudinal sections and partially transverse sections of muscle fibres are determined in most fields of view. The ratio of muscle to adipose tissue is 23/1.

The density of vessels is moderate, and single nerve trunks are clearly discernible. The density of the endomysial slits is within the normal range and contains a moderate amount of capillary lumen and fibroblast nuclei. The sarcoplasm is slightly thickened, with muscle fibre nuclei located parallel to the periphery of the longitudinal line of the muscle fibre. The transverse striation in the unaffected muscle fibres is preserved, and the myoglobin saturation is sufficient. The muscle fibres are thin. On histological section, the areas of lesion and the presence of five cysts are determined. In the affected muscle fibres, transverse striation is absent. Instead, the sarcoplasm forms a connective tissue capsule of an oval and round shape around the juvenile stage of cyst development. At the periphery of the cyst capsule, non-specific inflammation in the form of lymphohistiocytic infiltration with admixture of lymphocytes is identified. The degree of inflammation of muscle tissue at a distance from the lesion area is visualised in the form of focal serous myositis. Additional histochemical staining by Van Gizon revealed an increase in the density of fibrous fibres, which were observed to be concentrated in concentric structures on the periphery of neurovascular bundles and in the thickening of perimysium fibres embedded deep into endomysial spaces.

### 3.2. Molecular Analysis

**Figure 7:**
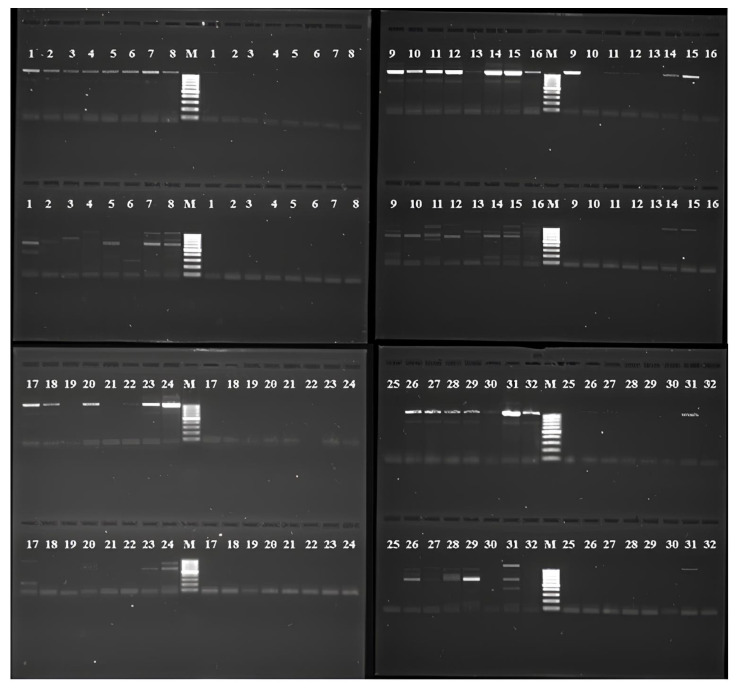
PCR results: A (SF1-SR9); B (SF1-SR4); C (SF1-SR5); D (SF1-COIRm); 1–23, amplified fragments; M, DNA marker 100–1000 bp.

Molecular genetic studies were conducted with the objective of identifying the species of *Sarcocystis* present in horses. A total of 32 sarcocysts were selected for the studies. The PCR products were not available for the primer pair SF1-CoxS1R. The positive PCR samples (Figure 7) were selected for sequencing.

In this study, partial sequences of the mitochondrial cytochrome c-oxidase (cox1) gene were obtained, and the results of molecular genetic analysis enabled the identification of 18 sarcocysts present in the muscles of horses from the Kostanai region (PP691482.1, PP691485.1, PP691486.1, PP691489.1, PP691491.1, PP691492.1, P691493.1, PP691494.1, PP691497.1, PP691501.1, PP691502.1, PP691503.1, PP691505.1, PP691483.1, PP691484.1, PP691496.1, PP691499.1, PP691506.1).

**Figure 8:**
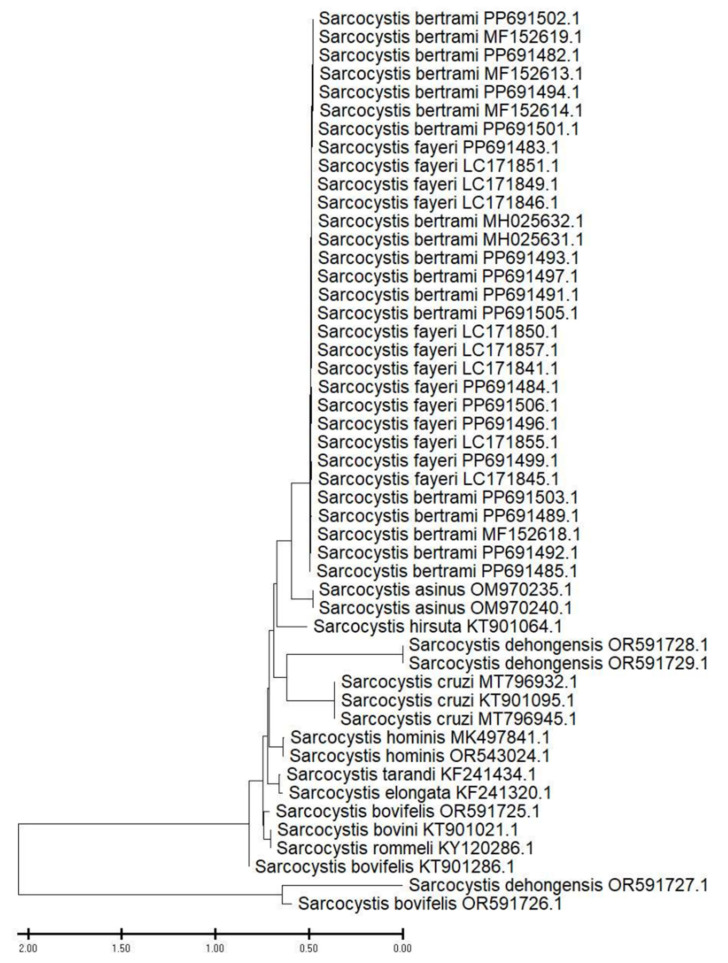
A phylogenetic tree of selected isolates of *Sarcocystis bertrami* and *Sarcocystis fayeri* found in horses in the Kostanai region and representative sequences deposited in GenBank based on partial cox1 sequences using the neighbour-joining method is presented. GenBank inventory numbers are indicated next to taxon names.

A comparison of the data obtained with reference sequences in databases enabled the identification of two parasite species and the potential for differences in horse infestations in different areas of the northern region to be identified. The phylogenetic analysis of *Sarcocystis* isolates yielded three distinct clades of *Sarcocystis bertrami* and two distinct clades of *Sarcocystis fayeri* (Figure 8). 

Forty-nine nucleotide sequences were involved in this analysis. Included codon positions were 1st + 2nd + 3rd + noncoding. All ambiguous positions were removed for each pair of sequences (pairwise deletion option). The pairwise patristic distances between sequences are presented in Table S1. The final dataset comprised a total of 733 positions (Table S1).

The four mitochondrial cox1 nucleotide sequences (PP691502.1, PP691482.1, PP691494.1, PP691501.1) were 724 bp in length and exhibited 99.31–99.45% identity with the mitochondrial cox1 nucleotide sequences of *Sarcocystis bertrami* from China (MF152619.1, MF152613.1, MF152614.1). The four mitochondrial cox1 nucleotide sequences (PP691493.1, PP691497.1, PP691491.1, PP691505.1) were 724 bp in length and exhibited 98.90–99.59% identity with the mitochondrial cox1 nucleotide sequences of *Sarcocystis bertrami* from China (MH025632.1, MH025631.1). A single mitochondrial cox1 nucleotide sequence (PP691483.1) was 724 bp in length and exhibited 99.45–99.72% identity with the mitochondrial cox1 nucleotide sequences of *Sarcocystis fayeri* from Japan (LC171851.1, LC171849.1, LC171846.1). The four mitochondrial cox1 nucleotide sequences (PP691484.1, PP691506.1, PP691496.1, PP691499.1) were 724 bp in length and exhibited 99.03–99.45% identity with the mitochondrial cox1 nucleotide sequences of *Sarcocystis fayeri* from Japan (LC171850.1, LC171857.1, LC171841.1, LC171855.1, LC171845.1). The four mitochondrial nucleotide sequences of cox1 (PP691503.1, PP691489.1, PP691492.1, PP691485.1) were 724 bp in length and exhibited 99.03–99.45% identity with the mitochondrial nucleotide sequence of cox1 of *Sarcocystis bertrami* from China (MF152618.1). This identity was confirmed by the GenBank database, which also corroborated the morphological identification.

The study of 132 horse carcasses revealed the presence of sarcocysts in 68 cases, corresponding to an invasion intensity of 51.5%. The research conducted on the territory of the Kostanay region revealed the presence of two species of sarcocysts in horses. The number of *Sarcocystis bertrami* was 13, while the number of *Sarcocystis fayeri* was 5.

## 4. Discussion

The results on the prevalence of infection in horses caused by sarcocystosis, as presented in the present work, are consistent with the findings of similar studies conducted globally. Sarcosporidiosis is a globally distributed disease affecting animals [5,6,7,8,9,10]. It is noteworthy that despite the extensive literature on sarcocystosis infection in domestic animals there is a paucity of research on sarcocystosis in horses.

In Kazakhstan, studies on the prevalence of sarcocystosis were conducted between the 1960s and 1980s. However, it was not until 2008 that data on the registration of sheep sarcocystosis in Western Kazakhstan emerged, with additional references of sarcocystosis in South-Eastern Kazakhstan [28]. In the northern region of the country, there is a dearth of information on animal sarcocystosis, which underscores the importance and necessity of conducting studies on the distribution of sarcocystosis in domestic animals. This study is the first to reveal the spread of equine sarcocystosis in northern Kazakhstan (Kostanay region), which is significant because it carries the risk of human infection, as the country has traditionally engaged in horse breeding for food [12].

The consumption of horse meat containing *Sarcocystis* has been linked to a number of reported cases of intestinal and muscular sarcocystosis worldwide, as evidenced by numerous studies [14,15,29,30,31]. It has been demonstrated that humans can act as both intermediate and definitive hosts for certain species of *Sarcocystis*. This is evidenced by studies conducted in Malaysia on the island of Tioman in the state of Pahang, where the largest cluster of symptomatic human muscular sarcocystosis was reported in 2011 and 2012 [14,15,16,30]. Human intestinal and muscular sarcocystosis is endemic in Southeast Asia, with previous reports from Thailand, Vietnam, the Lao People’s Democratic Republic (Lao PDR), and Malaysia [15,30]. The microscopic examination of faeces has revealed the presence of *Sarcocystis* parasites in humans in Iran (2017) [13] and Cambodia (2016) [31]. These parasites are characterised by an obligate two-host life cycle, with sarcocysts produced mainly in the muscles of intermediate hosts and endogenous sporulation of oocysts in the intestine of definitive hosts [30].

The findings of our research suggest a notable prevalence of infestation among horses in Kostanai. Consequently, 68 of the 132 horse carcasses examined exhibited evidence of sarcocysts, indicating an infestation prevalence of 51.5%. The prevalence of sarcocystis infection in horses varies considerably between countries and between studies conducted in different years. For example, high prevalence rates of infestation in horses have been reported in Brazilian and South American horses (100%, 51/51) [11], in Mongolia (up to 93%) [8], in China (up to 74%, 34/46) [23], and in British horses in 1981, where sarcocystis was detected in oesophageal samples in 62% of 394 horses and ponies [32]. The prevalence of infection in horses also exhibits considerable variation in Lithuania, with rates ranging from 34.7% to 63.9% [33], and in Japan, where the prevalence is 6% [30].

The frequency of infection was found to vary according to the type of muscle involved, with cardiac, smooth, and skeletal muscles all demonstrating differing susceptibility. The histological examination revealed that the sarcocysts were located within the muscle fibres, appearing as spindle-shaped inclusions. There was no evidence of transverse striation in the muscle fibres, and the sarcoplasm formed an oval-shaped connective tissue capsule. The dystrophic changes observed in equine muscle fibres are consistent with the findings of Russian scientists investigating the effects of sarcocystosis on bovine musculature [34]. In some cases, focal infiltrates from lymphocyte macrophages are observed, which indicates that the parasite has an immunogenic effect. A comparable prevalence of infection was observed in horses from Bahia state, as reported by Brazilian researchers in a previous study [11]. The aforementioned pathomorphological alterations in the proximate microscopic cysts of muscle tissue result in a reduction in the quality of meat products.

It can be observed that dystrophic changes occur in muscle fibres affected by sarcocysts, which serve as an indicator of toxic reactions. As stated by parasitologists, sarcocysts are known to produce sarcocystin, a substance with toxic effects, as well as other toxic substances that disrupt intracellular metabolism, sensitise the host organism, and stimulate the development of allergic reactions [17,35]. For example, studies were conducted in the Kumamoto Prefecture in Japan between 2009 and 2010 to determine whether *Sarcocystis* parasites could cause food poisoning in human consumers of raw horsemeat. The studies demonstrated that parasite toxins are implicated in the pathogenic mechanisms of parasitic food poisoning. Additionally, a recently identified protein, ADF, in the parasite *S. fayeri*, has been shown to function as a toxin that induces enteropathogenic changes and causes food poisoning in consumers of raw horsemeat [17].

Following molecular genetic analysis of 32 infected samples, only 18 samples passed the BLAST test. The data obtained were then compared with reference sequences in databases, which helped to determine the presence of two sarcocyst species in horse muscle: *Sarcocystis fayeri* and *Sarcocystis bertrami*. It was found that for both *Sarcocystis* species, dogs are the definitive host [36]. Additionally, possible differences in the infection of horses in different areas of the northern region have been identified.

Recent research has revealed that *Sarcocystis fayeri* and *Sarcocystis bertrami* are capable of infecting horses [23]. Two distinct species of *Sarcocystis* are known to infect horses: *S. fayeri*, a thick-walled species with a “type 11a” sarcocyst wall; and *S. bertrami*, a thin-walled species that is often considered synonymous with *S. equicanis*. The sarcocysts described here were diagnosed as *S. bertrami* based on their similarity to the “type 11c” sarcocysts previously demonstrated for *S. equicanis* from European horses [23,24,36]. A comparison and phylogenetic analysis revealed a close relationship between the the identified *Sarcocystis* species and other *Sarcocystis* equine DNA sequences from China. This was evidenced by the mitochondrial nucleotide sequence of cox1 of *Sarcocystis bertrami* (*MF152618.1*) and with the mitochondrial nucleotide sequences of cox1 *Sarcocystis fayeri* from Japan (*LC171850.1*, *LC171857.1*, *LC171841.1*, *LC171855.1*, *LC171845.1*), which the GenBank database corroborates with a morphological identification.

The prevalence of S. bertrami infection in equine tissues exhibits geographical variation, with the type of tissue examined and the diagnostic methods used contributing to this variability [10].

The results of this research demonstrate that two species of sarcocysts are present in horses within the Kostanay region. The number of *Sarcocystis bertrami* was 13, while the number of *Sarcocystis fayeri* was 5. The data were subsequently entered into the international database of genetic material, and identification numbers were generated. This is the inaugural histological and molecular study on the identification of *Sarcocystis* spp. isolated from microscopic forms of equine sarcocysts in the northern region of Kazakhstan.

## 5. Conclusions

For the first time in the territory of the Kostanay region, the prevalence of sarcocystosis among horses, both in adults and young animals, with an invasion intensity of 53.3%, was revealed.

Histological studies revealed the presence of inflammation of muscle tissue, which manifested as serous-reactive or chronic focal myositis. The affection of muscle fibres results in dystrophic changes, which are indicative of toxic reactions. The identified changes have a detrimental effect on the quality of meat products.

Genetic studies in horses have identified two species of *Sarcocystis*: *Sarcocystis bertrami* and *Sarcocystis fayeri*. The data on the distribution of equine sarcocystosis in the Kostanay region provide valuable information, which is a key step in the development of effective control and the prevention of this disease.

## Figures and Tables

**Table 1:** Oligonucleotide primers used in PCR.

DNA Region	Primer Name	Orientation	Primer Sequence (5′ to 3′)	Fragment Size
mtDNA cox1	SF1 SR9	Forward	ATGGCGTACAACAATCATAAAGAA	1085
		Reverse	ATATCCATACCRCCATTGCCCAT	
	SF1 SR4	Forward	ATGGCGTACAACAATCATAAAGAA	1060
		Reverse	CCACACCTGTAGTACCDCC	
	SF1 SR5	Forward	ATGGCGTACAACAATCATAAAGAA	1103
		Reverse	TAGGTATCATGTAACGCAATATCCAT	
	SF1 COIRm	Forward	ATGGCGTACAACAATCATAAAGAA	1142
		Reverse	CCCAGAGATAATACAAAATGGAA	
	SF1 CoxS1R	Forward	ATGGCGTACAACAATCATAAAGAA	1070
		Reverse	TTACCCATGACCACACCTGTAGTACC	

## Data Availability

All relevant data are presented within the paper.

## Supplementary Materials

The following supporting information can be downloaded at: https://www.mdpi.com/article/10.3390/ani14162299/s1, Table S1: Pairwise patristic distances between 49 nucleotide sequences.
